# APOE-ε4 Allele Altered the Rest-Stimulus Interactions in Healthy Middle-Aged Adults

**DOI:** 10.1371/journal.pone.0128442

**Published:** 2015-06-08

**Authors:** Feng-Xian Yan, Changwei W. Wu, Yi-Ping Chao, Chi-Jen Chen, Ying-Chi Tseng

**Affiliations:** 1 Department of Radiology, Taipei Medical University—Shuang-Ho Hospital, New Taipei City, Taiwan; 2 Department of Biomedical Sciences and Engineering, National Central University, Taoyuan, Taiwan; 3 Brain and Consciousness Research Center, Taipei Medical University, Taipei, Taiwan; 4 Graduate Institute of Medical Mechatronics, Chang Gung University, Taoyuan, Taiwan; Tianjin Medical University General Hospital, CHINA

## Abstract

The apolipoprotein E-ε4 allele is a well-known genetic risk factor for late-onset Alzheimer’s disease, which also impacts the cognitive functions and brain network connectivity in healthy middle-aged adults without dementia. Previous studies mainly focused on the effects of apolipoprotein E-ε4 allele on single index using task or resting-state fMRI. However, how these evoked and spontaneous BOLD indices interact with each other remains largely unknown. Therefore, we evaluated the ‘rest-stimulus interaction’ between working-memory activation and resting-state connectivity in middle-aged apolipoprotein E-ε4 carriers (n=9) and non-carriers (n=8). Four *n*-back task scans (*n* = 0, 1, 2, 3) and one resting-state scan were acquired at a 3T clinical MRI scanner. The working-memory beta maps of low-, moderate-, and high-memory loads and resting-state connectivity maps of default mode, executive control, and hippocampal networks were derived and compared between groups. Apolipoprotein E-ε4 carriers presented declined working-memory activation in the high-memory load across whole brain regions and reduced hippocampal connectivity compared with non-carriers. In addition, disrupted rest-stimulus interactions were found in the right anterior insula and bilateral parahippocampal regions for middle-aged adults with apolipoprotein E-ε4 allele. The rest-stimulus interaction improved the detectability of network integrity changes in apolipoprotein E-ε4 carriers, demonstrating the disrupted intrinsic connectivity within the executive-functional regions and the modulated memory-encoding capability within hippocampus-related regions.

## Introduction

The apolipoprotein E-ε4 (APOE-ε4) allele is a well-known genetic risk factor for late-onset Alzheimer’s disease (AD), causing symptoms after age 65 years [1]. The presence of the APOE-ε4 is associated with an swift decline in episodic memory [2] and visual attention [3], a decrease in neuronal development and synaptic plasticity [4], and increased risk of occurrence and earlier onset of AD in a gene dose-dependent manner [5]. Previous studies indicated that the APOE-ε4 allele exerts effects on the cognitive performance, including spatial attention and working memory in healthy middle-aged carriers without dementia [6].

Such irreversible cognitive deficits are supposed to originate from the regional alterations of brain functionality, which has been supported by neuroimaging literature. Morphology-based analysis using magnetic resonance imaging (MRI) revealed that APOE-ε4 carriers had greater cerebral atrophy in the hippocampus [7] and medial temporal lobe sub-regions [8]. Positron emission tomography study reported that asymptomatic APOE-ε4 carriers showed regional reductions of cerebral glucose metabolism qualitatively the same as clinical AD patients [9]. Functional MRI (fMRI) studies showed that age-variant APOE-ε4 carriers had increased or decreased activation within the brain regions using a variety of cognitive tasks. Compared with non-carriers, young APOE-ε3/ε4 carriers yielded robust bilateral frontoparietal activation using n-back working-memory (WM) tasks [10], and greater hippocampal activity during an encoding memory task [11]. Relative to young APOE-ε4 carriers, middle-aged and older APOE-ε4 carriers showed decreased activation in the temporal and cerebellar regions using an encoding memory task, suggesting that APOE-ε4 carriers fail to recruit normal age-related compensatory processes [12]. Besides, a previous study using a prospective memory task found middle-aged APOE-ε4 carriers had reduced activity in the extrastriate and partietal regions, along with an additional recruitment in the left frontal regions and left hippocampus; this is an indicative of a compensatory response [13]. In the healthy elderly APOE-ε4 carriers, decline in brain activation was found in the parietal and temporal regions during a visual WM task; whereas greater activation was exhibited in the prefrontal cortex, precentral and postcentral gyri, and parahippocampal gyrus relative to non-carriers [14]. Another literature using a verbal n-back WM tasks showed elderly APOE-ε4 carriers had increased activation in the frontal and parietal regions [15]. Based on previous literature [16], better execution of WM relies on the synergy of a set of mental capabilities including the central executive, phonological loop, and the visuospatial sketchpad. The central executive is important for control and manipulation of WM information by focusing and switching attention [17], which also plays an essential role in coordinating the operation of two other subsystems [16]. Several studies reported abnormal brain activation during WM tasks were found in subjects with mild cognitive impairment (MCI) and AD [17–19] as well as in APOE-ε4 carriers [6, 14, 15, 20], which may imply the importance of WM deficit in the initial stages of the disease process and might facilitate the early detection of AD [17]. Meanwhile, an alternative perspective beyond brain activation arises using so-called resting-state fMRI (RS-fMRI), in which the basal brain activity without task engagement is measured for estimating the brain network integrity (or functional connectivity) [21, 22]. It has been confirmed effective in disclosing functional abnormality in neurodegenerative pathologies or psychiatric disorders, such as AD [23] and major depression [24]. Using RS-fMRI, previous study showed that alterations in functional connectivity within the default mode network (DMN) and the executive control network (ECN) are present even before cognitive decline and brain atrophy in middle-aged APOE-ε4 carriers [5]. Additionally, disrupted hippocampal network (HPN) was also found in elderly APOE-ε4 carriers [25]. In summary, APOE-ε4 carriers demonstrated degradations of cerebral functionalities without explicit behavioral changes, indicating that the neuroimaging indices possess high sensitivity on functional abnormalities.

Beyond the high sensitivity across reported neuroimaging indices, the reciprocal individual diagnosis from these indices is ill-posed, implying the poor pathological specificity from single index. Previous studies mainly focused on investigating the effects of APOE-ε4 allele on the functional index using WM task- [14, 20] or RS-fMRI [5, 25]. However, how these neuroimaging indices interact with each other remains largely unknown. Based on Anand’s mismatched treatment effects for both task- and RS-fMRI on major depression patients, integrating multiple indices, especially for both evoked activation and resting connectivity, may serve a higher specificity to disease with the potential for early diagnosis [26]. Northoff et al. also supported the idea that the rest-stimulus interaction is relevant to predicting subsequent behavioral and mental states [27]. Henceforth, we tested whether the ‘rest-stimulus interactions’ serves a better predictor than single functional indices on APOE-ε4 carriers and non-carriers, when their cognition did not present between-group difference which may happen at mid-life. Based on previous studies, the antagonistic pleiotropy hypothesis proposed a transition point that APOE-ε4 allele exerts positive effects on cognition in young adulthood but negative effects in older adulthood [28], which suggest, plausibly, no effect of APOE-ε4 allele on cognition performance in middle-aged adults. Besides, differing from other literature using young or elderly populations, another reason we focused on middle-aged adults is that people in this age range possess less probability of onset of an AD prodrome [29]. The prodromal cognitive decline typical began on average 5–6 years prior to AD onset at mean age 77.4 years [30]. Therefore, we anticipate the possibility of early diagnosing APOE-ε4-associated cognition deterioration using the ‘rest-stimulus interactions’. To achieve this goal, we investigated the brain activation under WM tasks and the brain connectivity under a RS, and subsequently, correlated both imaging indices to explore the rest-stimulus interactions between middle-aged APOE-ε4 carriers and non-carriers (age- and gender-matched).

## Materials and Methods

### Ethics Statements

All participants declared fully understanding on the experimental procedure and signed written informed consents. The study was approved by the Institutional Review Boards of the Taipei Medical University.

### Participants

A total of one hundred ten participants underwent physical examinations at Taipei Medical University—Shuang-Ho Hospital. All participants were assessed with the Mini-Mental State Examination (MMSE) for an objective diagnosis of cognitive impairment by a neuropsychologist, and they also completed the Alzheimer’s Disease 8 (AD8) questionnaire for subjective cognitive impairment [31, 32]. Twenty-nine participants were excluded due to cognitive impairment. The rest of participants were enrolled into APOE genotyping. APOE genotyping for each patient was carried out as previously described [33], using amplification by polymerase chain reaction. After screening of APOE genotyping, twelve participants who were heterozygote (ε3/ε4) for APOE-ε4 and sixty-nine ε4-negative participants were examined. One participant with non-carrier was excluded due to image intensity inhomogeneity. Finally, data from the nine participants with APOE-ε4 carrier (5 females, age: 49.7 ± 13.2 yrs) and eight participants with APOE-ε4 noncarrier (5 females, age: 46.4 ± 12.0 yrs) were included in the final analysis.

### *N*-Back Working Memory Task

A total of 17 participants performed four numeral *n*-back task experiments (*n* = 0, 1, 2, 3) to assess the cognitive networks associated with working memory and attention. Two participants with APOE-ε4 carrier only performed three experimental conditions excluding 0-back condition. At the beginning of the formal experiment, a visual instruction describing the *n*-back task conditions and the rules of the experiment was projected to the screen, which can be viewed through a mirror set up above the head coil. After the visual instruction was shown, a sequence of Arabic numbers was subsequently presented on the screen. In the 0-back condition, participants were asked to read a stream of numerals and to judge if the emerging number matches the predefined target numeral. In other *n*-back conditions (*n* = 1, 2, 3), participants were asked to recall whether the presenting numeral matched the numeral presented *n* items before. All participants were instructed to press the left button with their right index finger at the ‘match’ condition, and to press the right button with their middle index finger at the ‘not match’ condition. Experimental presentation and behavioral scores (i.e., accuracy rate and response time) were collected and controlled by E-prime, the E-Studio software (Version 2.0; Psychology Software Tools, Inc.; Pennsylvania, USA; http://www.pstnet.com/eprime.cfm).

In each experimental condition, we utilized a simple block design with four resting epochs and three active task epochs. All epochs lasted for 30 seconds, and the total duration was 210 seconds. In the resting epochs, participants were instructed to focus on a fixation cross positioned at the center of the screen. In the active task epochs, each trial consisted of an Arabic number (among 0 to 9) for 0.5 seconds, and a fixation cross lasted for 2 seconds. The inter-trial interval was 2.5 seconds, and the total number of trials was 36. The entire WM tasks were about 15 min. Except MRI recordings, accuracy rates and response times were recorded for all *n*-back task experiments (*n* = 0, 1, 2, 3).

### MRI Acquisition

Imaging data were acquired using a 3 Tesla GE whole-body scanner (Discovery MR750; GE Healthcare Systems, Milwaukee, Wisconsin), equipped with an 8-channel receive-only head coil. During the WM task-fMRI scan, participants were instructed to perform four *n*-back WM tasks (*n* = 0, 1, 2, 3). The functional images were acquired parallel to the anterior–posterior commissure plane using a T_2_*-weighted single-shot gradient-echo echo-planar imaging (EPI) sequence with the following parameters: repetition time (TR) / echo time (TE) = 3000 ms / 35 ms, flip angle (FA) = 90°, field of view (FOV) = 230 × 230 mm^2^, in-plane matrix = 64 × 64, slice thickness = 3 mm with 1 mm gap. For each participant, 40 axial slices per volume and a total of 70 volumes were obtained. Two dummy scans were applied to achieve a steady state. During the RS-fMRI scan, participants lay supine in the scanner with their head positions fixed with cushions. They were instructed to relax, to keep their eyes closed, to stay awake, to think of nothing, and to perform no specific tasks. The functional images were acquired with the same positions as the task-fMRI scan using a T_2_*-weighted single-shot gradient-echo EPI sequence with the following parameters: TR / TE = 2000 ms / 30 ms, FA = 90°, FOV = 230 × 230 mm^2^, in-plane matrix = 64 × 64, and slice thickness = 3 mm with 1 mm gap, 40 axial slices, 320 volumes, and 2 dummy scans. After scanning, all participants reported that they did not fall asleep during whole fMRI scans.

### Data Analysis

#### Data Preprocessing

All functional images including four WM task-fMRI scans and one RS-fMRI scan were preprocessed using SPM8 (Statistical Parametric Mapping 8; Wellcome Department of Imaging Neuroscience, London, UK; http://www.fil.ion.ucl.ac.uk/spm/). Data preprocessing included slice-timing correction, realignment for head-motion correction, spatial normalization to the Montreal Neurologic Institute (MNI) template, and spatial smoothing with a 6-mm Gaussian kernel. For all fMRI scans and all participants, the head motion levels were controlled less than 3-mm in translation and less than 3° in rotation.

#### WM Task Analysis

In the first-level individual analysis, the smoothed images of each *n*-back task were separately analyzed for each individual participant. For each *n*-back task experiment, the time course of experimental condition was modeled by convolving a boxcar function representing the times of onset and duration of each stimulus block with a canonical hemodynamic response function using SPM8. Six head-motion parameters from realignment were modeled as nuisance regressors. General linear model was used to evaluate the task-related activity. Statistical contrast was conducted to compare the active task vs. resting. In the second-level within-group analysis, three WM contrast maps: low-memory load (1-back > 0-back), moderate-memory load (2-back > 1-back), and high-memory load (3-back > 2-back), from the individual participant analysis were analyzed by one-sample *t* tests. All statistical maps were set at a level of an uncorrected significance threshold of *p* < 0.05 and a cluster threshold = 740 voxels. This yielded a significance of *p* < 0.05, corrected for multiple comparisons, determined by the AlphaSim program [34].

#### RS Functional Connectivity Analysis

The smoothed images of RS-fMRI scan were then processed using REST (Resting-State fMRI Data Analysis Toolkit; http://www.restfmri.net [34]) including removal of linear trends and temporal band-pass filtering (0.01–0.08 Hz) to reduce the effects of low-frequency drift and high-frequency physiological noise. Spurious signals, including six head-motion parameters from realignment and other signals within the global, white matter, and cerebrospinal fluid masks, were regressed out. Three different seed regions with 3-mm radius were defined based on previous literature, including the posterior cingulate cortex (-1, -50, 26) for DMN [5], the right dorsolateral prefrontal cortex (44, 36, 20) for ECN [5], and the bilateral hippocampus regions (±24, -22, -20) for HPN [35]. These three cognitive resting-state networks (RSNs) are greatly associated with memory and attention: the DMN is associated with episodic memory retrieval; the ECN is involved in goal-directed behavior and cognitive control; and the hippocampus, which is one of the first regions to show AD-related pathology [36], plays an important role in regulating high-order cognitive function, explicit memory and memory consolidation. Except the seed within the bilateral hippocampus, other coordinates of seed regions were defined in the Talairach space [37], and converted to the MNI coordinates using an algorithm implemented in the MATLAB script (tal2icbm_spm.m, http://brainmap.org/icbm2tal/) for further analysis [38].

Functional connectivity analysis was performed on each participant and each seed using REST, the reference time course was extracted by averaging the time courses of all voxels in each seed. The functional connectivity map was obtained by computing Pearson's correlation coefficient (r) between the reference time course and the time course from every other voxel in the brain. The r map was then transformed to an approximate Gaussian distribution using Fisher’s z transformation. For each RSN, a one-sample *t* test was performed on the z maps to identify the voxels with significant correlations with each seed (for DMN: *p* < 0.01, corrected for multiple comparisons with false discovery rate (FDR) criterion, cluster threshold = 10 voxels; for ECN and HPN, *p* < 0.05, corrected with FDR, cluster threshold = 10 voxels).

#### Region of Interest (ROI) Analysis

To quantify index differences between both groups, we applied the ROI analysis to calculate the average beta values in three WM contrast maps (i.e., low-, moderate-, and high-memory loads), and the average z scores in three RS connectivity maps (i.e., DMN, ECN, and HPN). To generate the target ROIs without the group bias, we performed the group independent component analysis (ICA) using GIFT (Group ICA of fMRI Toolbox; Medical Image Analysis Lab, University of New Mexico, Albuquerque, New Mexico; http://icatb.sourceforge.net) to obtain the DMN and ECN ROIs. The smoothed images of RS-fMRI scan for all participants (n = 17) were temporally concatenated into a single 4-dimension data set, and then analyzed by two-step principal component analysis, followed by independent component estimation with the Infomax algorithm [39]. The group ICA was performed 20 times using the ICASSO toolbox [40] to ensure stability and reliability of the independent component decomposition [41]. Twenty-five independent component spatial maps were extracted to describe the large-scale patterns of RSNs across the participants. A set of average group components were created and back-reconstructed into the native space [39]. The individual participants’ components were scaled to spatial z-score maps by subtracting the global mean from each voxel and divided by the global standard deviation [39]. In each component, a one-sample *t* test was performed on the scaled z maps (*p* < 0.05, corrected for multiple comparisons with family-wise error criterion, cluster threshold = 10 voxels). Afterward, these *t* maps of each component were visually inspected and selected as the ICA-derived DMN and ECN (i.e., left and right frontoparietal networks) templates. Because the group ICA could not clearly identify the HPN, the HPN regions were defined based on the automated anatomical labeling (AAL) template [42], including bilateral hippocampal and bilateral parahippocampal regions.

Quantitative analysis was carried out for each participant using MarsBar toolbox (MARSeille Boîte À Région d'Intérêt, http://marsbar.sourceforge.net/ [43]) within six DMN, twelve ECN, and four HPN regions. Without any outliers were excluded from ROI analysis using criteria within mean ± 3 standard deviation. The significant between-group comparisons were determined based on a statistical threshold of two-tailed, uncorrected *p* < 0.05.

#### Correlations between RS Connectivity and WM Activation

To explore the rest-stimulus interaction in APOE-ε4 carriers and non-carriers, the correlation coefficients were calculated between RS connectivity results (i.e., DMN, ECN, and HPN) and WM activation results (i.e., low-, moderate-, and high-memory loads) within the specific ROIs with significant between-group differences.

## Results

### Demographic and Behavioral Data

Table 1 summarizes the demographic data, neuropsychological and behavioral scores of the *n*-back tasks (*n* = 0, 1, 2, 3) in APOE-ε4 carriers and non-carriers. A two-sample *t* test was used to compare the differences in the demographic and behavioral data. There were no significant between-group differences (two-tailed, *p* > 0.05) in age, neuropsychological scales (i.e., MMSE and AD8 scores), and behavioral scores (i.e., accuracy rates and response times in all task experiments).

**Table 1:** Demographic data, neuropsychological and behavioral scores of APOE-ε4 carriers and non-carriers.

		APOE-ε4 carriers (n = 9)	APOE-ε4 non-carriers (n = 8)	*p*-value
Gender, Male / Female		4 / 5	3 / 5	-
Age, year		49.7 ± 13.2	46.4 ± 12.0	0.60
MMSE score		28.9 ± 1.17	29.6 ± 0.74	0.15
AD8 score		0.1 ± 0.33	0.6 ± 0.74	0.08
n-back accuracy rate (%)				
	0-back	89.7 ± 16.6	92.4 ± 12.7	0.36
	1-back	94.3 ± 6.0	90.9 ± 16.4	0.29
	2-back	82.6 ± 10.9	89.6 ± 10.1	0.10
	3-back	82.3 ± 9.8	89.8 ± 12.2	0.09
n-back response time (msec)				
	0-back	685.1 ± 139.4	659.4 ± 65.1	0.32
	1-back	856.7 ± 187.0	795.0 ±43.3	0.19
	2-back	1033.0 ± 194.3	1022.6 ± 214.2	0.46
	3-back	1008.9 ± 184.0	987.9 ± 154.8	0.40

MMSE = Mini-Mental State Examination; AD8 = Alzhimer’s Disease 8.

### Group Analysis in APOE-ε4 Carriers and APOE-ε4 Non-Carriers

**Fig 1:**
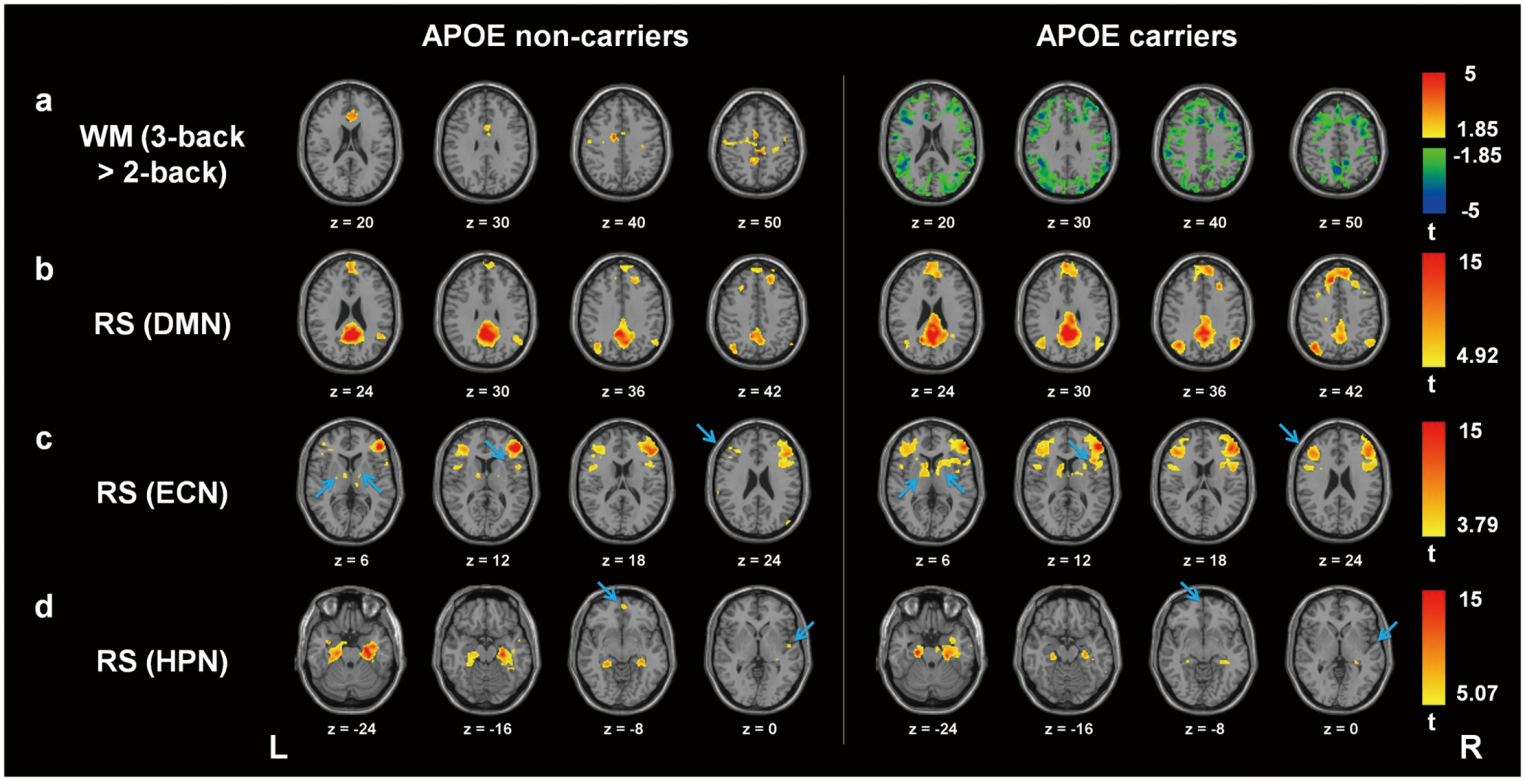
Group analysis in APOE-ε4 carriers and non-carriers. One-sample *t* test results of (a) WM activation in the high-memory load (3-back > 2-back), RS connectivity of (b) DMN, (c) ECN, and (d) HPN in APOE-ε4 carriers and non-carriers. (a) APOE-ε4 carriers showed decreased activation across the whole brain regions. (b) Both groups displayed consistent DMN connectivity maps. (c) APOE-ε4 carriers displayed enhanced ECN connectivity in the left middle frontal gyrus, right insula, and bilateral thalamus (blue arrows). (d) APOE-ε4 carriers showed decreased HPN connectivity in the medial frontal gyrus and right insula (blue arrows). WM = working-memory; RS = resting-state; DMN = default mode network; ECN = executive control network; HPN = hippocampal network; L = left hemisphere; R = right hemisphere.

In general, consistent and similar WM patterns were found in the *n*-back tasks (*n* = 0, 1, 2, 3) between APOE-ε4 carriers and non-carriers. Consistent with previous study [20], APOE-ε4 carriers had greater effect size and spatial extent than non-carriers in the low-memory load. In contrast, APOE-ε4 carriers showed less increments of spatial extent than non-carriers in the moderate- and high-memory load [20]. Fig 1a shows the one-sample *t* test results of WM activation in the high-memory load (3-back > 2-back) in APOE-ε4 carriers and non-carriers. APOE-ε4 non-carriers displayed increased activation in the anterior cingulate cortex, bilateral postcentral gyrus, left superior temporal gyrus, and left insula (Fig 1a, left); whereas APOE-ε4 carriers showed decreased activation across the whole brain regions, especially in the WM regions (Fig 1a, right).

Fig 1b shows the one-sample *t* test results of DMN connectivity in APOE-ε4 carriers and non-carriers. Consistent with previous study, both groups showed similar DMN connectivity patterns, including the left superior medial frontal gyrus and bilateral angular guri. Fig 1c displays the one-sample *t* test results of ECN connectivity in both groups. Compared with non-carriers, APOE-ε4 carriers had enhanced ECN connections in the left middle frontal gyrus, right insula, and bilateral thalamus (blue arrows). Fig 1d shows the one-sample *t* test results of HPN connectivity in both groups. Compared with non-carriers, APOE-ε4 carriers had decreased HPN connectivity in the medial frontal gyrus and the right insula (blue arrows).

### Rest-Stimulus Interactions in APOE-ε4 Carriers and APOE-ε4 Non-Carriers

To explore the rest-stimulus interactions in APOE-ε4 carriers and non-carriers, we firstly measured the average beta values (i.e., WM activation of the low-, moderate-, and high-memory load) and the average z scores (i.e., RS connectivity of the DMN, ECN, and HPN) in the memory/attention-associated regions for both groups. The selected regions were based on the ICA-derived DMN and ECN templates and the AAL-based HPN regions. The correlation coefficient was then computed between the WM activation index and the RS connectivity index. Tables 2–4 summarize the quantitative results, including average beta values and average z scores, in APOE-ε4 carriers and non-carriers within the ICA-derived DMN, ICA-derived ECN, and AAL-based HPN regions.

**Table 2:** Quantitative results, including the average beta values (i.e., high-memory-load activation) and the average z scores (i.e., DMN connectivity), within the ICA-derived DMN regions in APOE-ε4 carriers and non-carriers.

		Average beta values		Average z scores	
Region	Side	APOE-ε4 carriers	APOE-ε4 non-carriers	APOE-ε4 carriers	APOE-ε4 non-carriers
Superior Frontal Gyrus	L	-0.35 ± 0.44	0.05 ± 0.4	0.32 ± 0.14	0.34 ± 0.19
	R	-0.32 ± 0.36	0.09 ± 0.37*	0.28 ± 0.19	0.26 ± 0.15
Middle Temporal Gyrus	L	-0.47 ± 0.62	0.08 ± 0.53	0.48 ± 0.14	0.39 ± 0.22
	R	-0.35 ± 0.51	0.05 ± 0.91	0.42 ± 0.16	0.32 ± 0.17
Precuneus	R	-0.19 ± 0.44	0.14 ± 0.34	0.54 ± 0.11	0.48 ± 0.12
Anterior Cingulate	R	0.1 ± 0.82	0.19 ± 1.05	0.38 ± 0.14	0.27 ± 0.15

*: *p* < 0.05, compared with APOE-ε4 carriers.

DMN = default mode network; ICA = independent component analysis; L = left hemisphere; R = right hemisphere.

**Table 3:** Quantitative results, including the average beta values (i.e., high-memory-load activation) and the average z scores (i.e., ECN connectivity), within the ICA-derived ECN regions in APOE-ε4 carriers and non-carriers.

		Average beta values		Average z scores	
Region	Side	APOE-ε4 carriers	APOE-ε4 non-carriers	APOE-ε4 carriers	APOE-ε4 non-carriers
Medial Frontal Gyrus	L	-0.37 ± 0.55	0.02 ± 0.66	0.23 ± 0.13	0.18 ± 0.19
Middle Frontal Gyrus	L	-0.41 ± 0.38	0.06 ± 0.43*	0.2 ± 0.09	0.17 ± 0.11
Inferior Frontal Gyrus	L	-0.74 ± 0.69	0.16 ± 0.74*	0.43 ± 0.21	0.36 ± 0.34
Precentral Gyrus	R	-0.38 ± 0.42	-0.02 ± 0.65	0.35 ± 0.16	0.29 ± 0.16
Superior Temporal Gyrus	R	-0.28 ± 0.67	-0.12 ± 0.88	0.33 ± 0.15	0.25 ± 0.2
Middle Temporal Gyrus	L	-0.5 ± 0.51	0.08 ± 0.42*	0.09 ± 0.17	0.22 ± 0.21
	R	-0.16 ± 0.6	-0.07 ± 0.6	0.12 ± 0.3	0.24 ± 0.24
Angular Gyrus	L	-0.48 ± 0.83	0.04 ± 0.57	0.14 ± 0.14	0.18 ± 0.19
Inferior Parietal Lobule	L	-0.56 ± 1.28	0.1 ± 0.72	0.18 ± 0.17	0.21 ± 0.2
Cingulate Gyrus	L	-0.2 ± 0.74	0.33 ± 0.66	-0.01 ± 0.11	0 ± 0.15
Anterior insula	L	-0.17 ± 0.17	0.13 ± 0.45	0.18 ± 0.13	0.03 ± 0.13*
	R	-0.26 ± 0.37	0.06 ± 0.47	0.28 ± 0.21	0.05 ± 0.14*

*: *p* < 0.05, compared with APOE-ε4 carriers.

ECN = executive control network; ICA = independent component analysis; L = left hemisphere; R = right hemisphere.

**Table 4:** Quantitative results, including the average beta values (i.e., high-memory-load activation) and the average z scores (i.e., HPN connectivity), within the AAL-based HPN regions in APOE-ε4 carriers and non-carriers.

		Average beta values		Average z scores	
Region	Side	APOE-ε4 carriers	APOE-ε4 non-carriers	APOE-ε4 carriers	APOE-ε4 non-carriers
Hippocampus	L	-0.17 ± 0.32	0.05 ± 0.38	0.18 ± 0.07	0.24 ± 0.1
	R	-0.09 ± 0.27	0 ± 0.35	0.2 ± 0.08	0.27 ± 0.06
ParaHippocampal	L	-0.11 ± 0.42	-0.05 ± 0.43	0.27 ± 0.11	0.44 ± 0.13*
	R	-0.1 ± 0.34	-0.05 ± 0.4	0.33 ± 0.11	0.43 ± 0.08

*: *p* < 0.05, compared with APOE-ε4 carriers.

HPN = hippocampal network; AAL = automated anatomical labeling; L = left hemisphere; R = right hemisphere.

**Fig 2:**
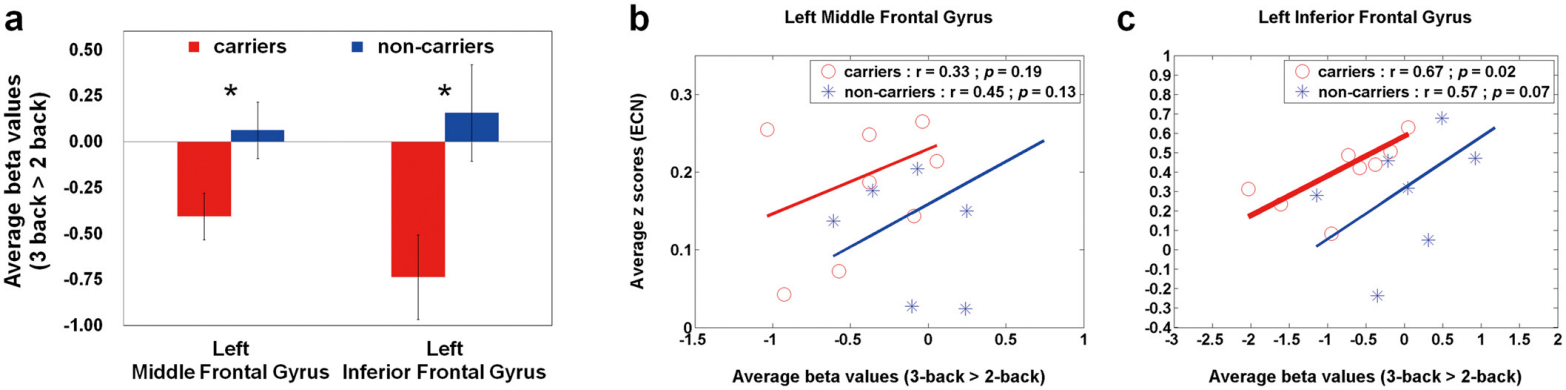
Quantitative analysis (average beta values) and Rest-stimulus interactions (ECN connectivity vs. high-memory-load activation) in APOE-ε4 carriers and non-carriers. (a) A bar graph of the average beta values (3-back > 2-back) in both groups within the specific ROIs, where the error bar stands for the standard error. APOE-ε4 carriers showed decreased working-memory activations within the left middle and inferior frontal gyri. The scatter plots of working-memory activation (3-back > 2-back) versus resting-state connectivity (ECN) within the (b) left middle frontal gyrus and (c) left inferior frontal gyrus in both groups. Both groups had similar positive trend between both neuroimaging indices. The significantly correlation was only found in APOE-ε4 carriers within the left inferior frontal gyrus. ECN = executive control network.

For WM activation patterns, there are no significant between-group differences in the low- and moderate-memory loads, except in the high-memory load. Fig 2a displays the average beta values of the high-memory load (3-back > 2-back) in APOE-ε4 carriers and non-carriers. Compared with non-carriers, APOE-ε4 carriers showed significantly lower average beta values within the left middle frontal gyrus (*p* = 0.03) and left inferior frontal gyrus (*p* = 0.02). Based on the two ROIs, Fig 2b and 2c illustrate the rest-stimulus interactions within the left middle frontal gyrus and left inferior frontal gyrus for both groups, respectively. These scatter plots exhibited that both groups had similar positive trend between ECN connectivity and high-memory-load activation. The significantly rest-stimulus interaction was only found in APOE-ε4 carriers within the left inferior frontal gyrus (r = 0.67, *p* = 0.035). Combined with Fig 2a, it was noteworthy that APOE-ε4 carriers presented significantly lower WM activation but similar ECN connectivity in these two ROIs compared with non-carriers (Table 2). Additionally, the significantly reduced beta values were also found in the right superior frontal gyrus and left middle temporal gyrus (Table 2), that showed no significantly rest-stimulus interactions for each group.

**Fig 3:**
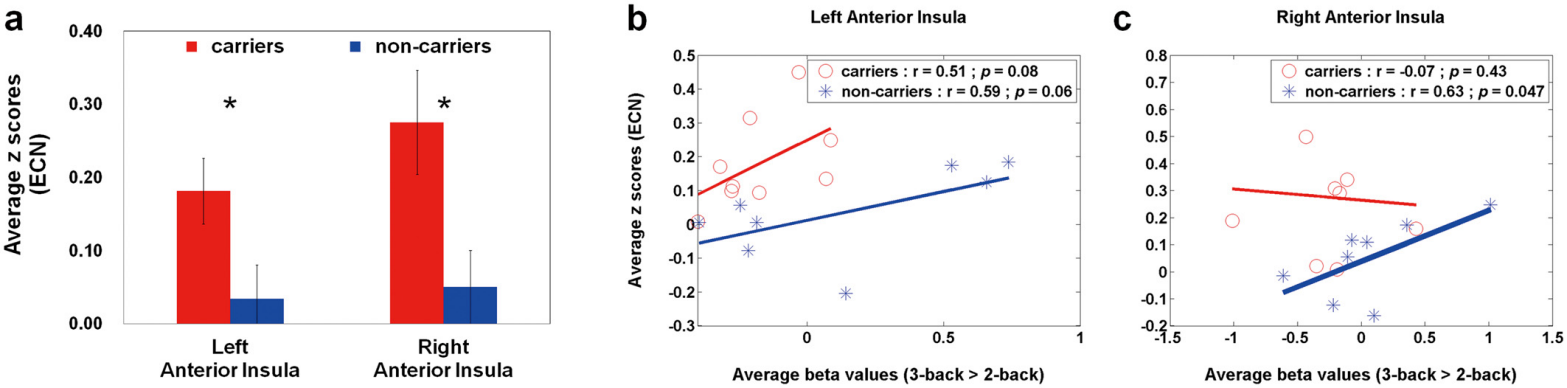
Quantitative analysis (average z scores) and Rest-stimulus interactions (ECN connectivity vs. high-memory-load activation) in APOE-ε4 carriers and non-carriers. (a) A bar graph of the average z scores (ECN) in both groups within the specific ROIs, where the error bar stands for the standard error. APOE-ε4 carriers had enhanced ECN connections in the bilateral anterior insula. The scatter plots of working-memory activation (3-back > 2-back) versus resting-state connectivity (ECN) within the (b) left and (c) right anterior insula in both groups. The significantly positive correlation was only found in APOE-ε4 non-carriers within the right anterior insula. ECN = executive control network.

**Fig 4:**
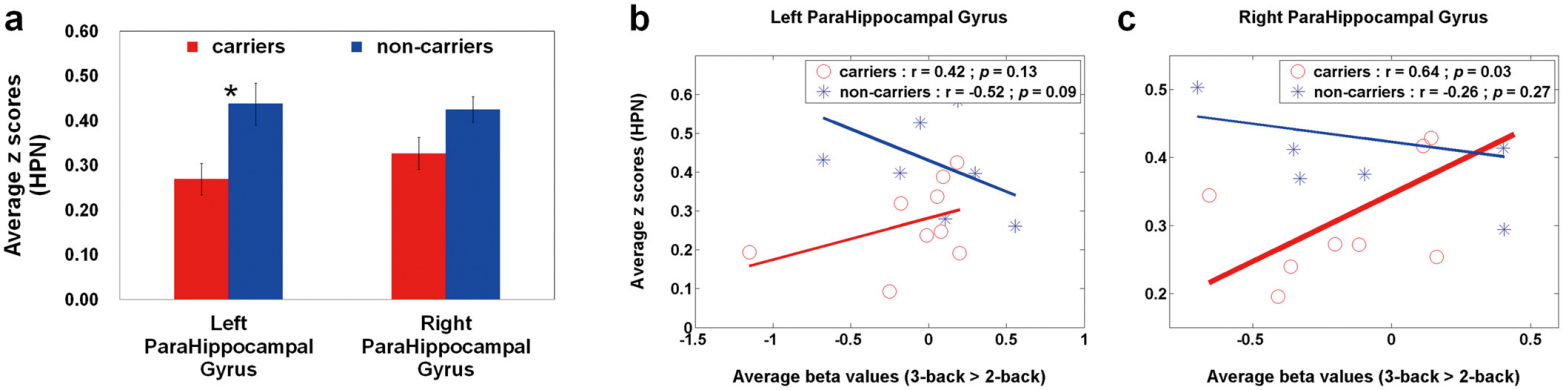
Quantitative analysis (average z scores) and Rest-stimulus interactions (HPN connectivity vs. high-memory-load activation) in APOE-ε4 carriers and non-carriers. (a) A bar graph of the average z scores (HPN) in both groups within the specific ROIs, where the error bar stands for the standard error. APOE-ε4 carriers had decreased HPN connections in the bilateral parahippocampal regions, especially in the left parahippocampal gyrus. The scatter plots of working-memory activation (3-back > 2-back) versus resting-state connectivity (HPN) within the (b) left and (c) right parahippocampal gyri in both groups. APOE-ε4 non-carriers presented negative trend within both regions; whereas APOE-ε4 carriers showed positive trend within both regions. The significantly positive correlation was only found in APOE-ε4 carriers within the right parahippocampal gyrus. HPN = hippocampal network.

For RS connectivity patterns, there are no significant between-group differences in the ICA-derived DMN ROIs, except in the ICA-derived ECN ROIs and AAL-based HPN ROIs. Fig 3a shows the average z scores of the ECN in APOE-ε4 carriers and non-carriers. Compared with non-carriers, APOE-ε4 carriers had enhanced ECN connections in the bilateral anterior insula (left: *p* = 0.04; right: *p* = 0.02). Based on the two ROIs, Fig 3b and 3c demonstrates the rest-stimulus interactions within the bilateral anterior insula for both groups, respectively. These scatter plots displayed that the significantly positive trend was only found in APOE-ε4 non-carriers within the right anterior insula (r = 0.63, *p* = 0.047). Fig 4a shows the average z scores of the HPN in APOE-ε4 carriers and non-carriers. Compared with non-carriers, APOE-ε4 carriers had decreased HPN connectivity in the bilateral parahippocampal regions, especially in the left parahippocampal gyrus (*p* = 0.01). Based on the two ROIs, Fig 4b and 4c illustrates the rest-stimulus interactions within the bilateral parahippocampal gyri for both groups, respectively. These scatter plots demonstrated that both groups had inconsistent trend between HPN connectivity and high-memory-load activation. APOE-ε4 non-carriers presented negative trend within both regions; whereas APOE-ε4 carriers showed positive trend within both regions. The significantly positive correlation was only found in APOE-ε4 carriers within the right parahippocampal gyrus (r = 0.64, *p* = 0.04).

## Discussion

Both group results and ROI analysis showed that APOE-ε4 carriers possess diminished WM activation in the high-memory load across whole brain regions, especially in the left middle and inferior frontal gyri, as well as present reduced HPN connectivity strength and spatial extent in the left parahippocampal gyrus relative to APOE-ε4 non-carriers. Integrating both neuroimaging indices, our results demonstrated the disrupted rest-stimulus interactions in the right anterior insula and the bilateral parahippocampal regions for APOE-ε4 carriers, implying that the rest-stimulus interactions indeed serves a better predictor than single imaging index.

An integrated WM model, which consists of central executive, phonological loop, and visuospatial sketchpad, was applied to clinical studies [16]. These studies demonstrated altered brain activations associated with WM and suggested the recruitment of compensatory mechanism in patients with AD or at risk of AD [17–19]. Consistent with previous studies, APOE-ε4 carriers had abnormal WM activation patterns in the high-memory load with similar accuracy rates and response times in the 2-back and 3-back WM tasks. Based on previous literature [20], mild impairment of WM capacity for APOE-ε4 carriers may probably explain this phenomenon. In middle-aged adults with APOE-ε4 allele, they recruited greater additional resources to compensate for processing inefficiencies in the low-memory load as well as had already recruited most of their (assuming a finite amount) available resources in the moderate- and high-memory loads. However, few studies had focused on the WM activation in the higher memory-load experiments (i.e., 3-back WM task), because these experiments were comparatively difficult for elderly participants [44].

It is noteworthy that APOE-ε4 carriers had impaired local connectivity between hippocampus and parahippocampal regions, especially in the left parahippocampal gyrus. Consistent with previous studies, intrinsic connectivity in HPN are disrupted across different cortical areas in middle-aged [45], elderly APOE-ε4 carriers [25], and elderly AD group [46]. These findings suggest that the functional disconnection between hippocampus and other cortical areas may reflect a breakdown of hippocampus-related networks in pre-symptomatic APOE-ε4 carriers and AD patients.

The unexpected finding was that, APOE-ε4 carriers presented stronger ECN connections to bilateral anterior insula. The anterior insula, a hub of the salience network (SN), plays an important role in mediating dynamic interactions between the RSNs involved in internally oriented cognition (e.g., DMN) and the RSNs involved in externally oriented attention (e.g., ECN) [47]. Based on previous studies, the enhanced SN connectivity co-occurs with the decreased DMN connectivity in healthy elderly APOE-ε4 carriers [48] and in patient with mild AD [49]. Moreover, disrupted insula connectivity was associated with episodic memory deficits in amnestic MCI patients [50]. The results suggested the contribution of abnormal insula connectivity to the cognitive deterioration in the pre-dementia stage.

The positive rest-stimulus interactions between ECN connectivity and high-memory-load activation were demonstrated in the left middle and inferior frontal gyri for both groups. APOE-ε4 carriers involved lower neural activity and/or fewer cortical areas in both regions when performing higher memory-load task, but they maintained the resembling behavioral performance and ECN connectivity relative to non-carriers. One possible speculation is that APOE-ε4 allele may induce the disruption of intrinsic connectivity and network integrity within the executive-functional regions in midlife. These impaired regions at rest could progressively impact the regional brain activity at the most difficult task level. Although the severity levels of abnormal brain connections had not seen in clinical at the present time, the impaired intrinsic connectivity might result in the decreased WM accuracy in older APOE-ε4 carriers [29], and the incremental additional cortical areas with widespread reduction of neural activity in older APOE-ε4 carriers [14].

The rest-stimulus interactions between HPN connectivity and high-memory-load activation were inconsistent between groups. APOE-ε4 non-carriers showed negative trend; whereas APOE-ε4 carriers presented positive trend. Both groups displayed the reverse rest-stimulus interactions in the bilateral parahippocampal regions, even though they had resembling behavioral performance and brain activity in the higher memory-load task. These results indicated that, compared with non-carriers, APOE-ε4 carriers required more resources outside the HPN (connectivity) and greater hippocampus-connected regions at rest to accomplish the WM mediation and long-term memory encoding [51], that are the important functions for parahippocampal gyri, at the most difficult task level. This finding implied that lower local connections in hippocampus-parahippocampal regions at rest may lead to poor memory encoding and retrieval, as well as memory deficits or decline in old age.

Based on previous studies, APOE-ε4 carriers showed increased DMN coactivation in young adults [11], but reduced DMN connectivity in middle-aged [5] and elderly [48]. Integrating these findings, age-related alterations of DMN connection might occur in adults with an APOE-ε4 allele. Nevertheless, in our study, the resembling DMN connectivity patterns between middle-aged APOE-ε4 carriers and non-carriers were shown in either group or ROI analysis, which was contradictory with previous report [5] using the same seed within the posterior cingulate cortex. Except using different population, the major difference is that on average, our population was slightly younger than their sample. One potential explanation for these inconsistent results is the age-related transition of DMN connection in our sample.

Several limitations existed in our present study. First, both populations had relatively small sample sizes. The main rationale is that the prevalence of APOE-ε4 allele in healthy normal controls is lower in Asian (15.5% of ε3/ε4 and 0.8% of ε4/ε4) than in Caucasian (21.3% of ε3/ε4 and 1.8% of ε4/ε4) [1]. In our study, the occurring frequency of APOE-ε4 allele with heterozygote (ε3/ε4) is about 13.5%, close to 15.5% [1]. Additionally, middle-age is seldom reported since the current literature focuses on young or elderly populations. However, it is not an easy task to recruit healthy middle-aged participants, because normal controls in the middle age are typically lack of time in participating studies in Taiwan. Although the sample size in this study was relatively small (17 effective out of 110 screened participants), our results of ROI analysis and correlations both reached statistical significances (*p* < 0.05). Second, the effect of β-amyloid pathology might have potential influences on our findings. Numerous studies have evidenced that β-amyloid burden might occur many years before objective or subjective cognitive declines, especially among APOE-ε4 carriers [52, 53]. Thus, the large-scale research investigations using β-amyloid imaging are warranted to confirm the current results.

## Conclusion

This is the first study to comprehensively explore the rest-stimulus interaction in middle-aged APOE-ε4 carriers and non-carriers (age- and gender-matched) using both WM task- and RS-fMRI. For each functional index, APOE-ε4 carriers possess abnormal cerebral functionalities without explicit behavioral changes in contrast to APOE-ε4 non-carriers. Our results demonstrated that the rest-stimulus interaction improved the detectability of network integrity changes in APOE-ε4 carriers, confirming the potentiality of adopting ‘rest-stimulus interactions’ on early prognosis of cognition deterioration caused by APOE-ε4 allele.
